# Effect of Plasma-Activated Water Pretreatment Combined with High-CO_2_ Modified Atmosphere Packaging on the Quality and Microbial Profile of Half-Smooth Tongue Sole (*Cynoglossus semilaevis*) During Superchilling Storage

**DOI:** 10.3390/foods15030529

**Published:** 2026-02-03

**Authors:** Xiang Qiu, Jun Mei, Jing Xie

**Affiliations:** 1 College of Food Science and Technology, Shanghai Ocean University, Shanghai 201306, China; m240451273@st.shou.edu.cn; 2 National Experimental Teaching Demonstration Center for Food Science and Engineering, Shanghai Ocean University, Shanghai 201306, China; 3 Shanghai Engineering Research Center of Aquatic Product Processing and Preservation, Shanghai 201306, China; 4 Shanghai Professional Technology Service Platform on Cold Chain Equipment Performance and Energy Saving Evaluation, Shanghai 201306, China

**Keywords:** high-CO_2_ system, protein oxidation, shelf life, preservation, autolysis and amine formation

## Abstract

Half-smooth tongue sole has high nutritional value due to its delicious meat and high protein content. However, its high protein content makes it highly susceptible to spoilage caused by microbial action. This study utilized plasma-activated water to pretreat half-smooth tongue sole, which was then subjected to various packaging methods: CK (air packaging), VP (vacuum packaging), MAP1 (75% CO_2_/5% O_2_/20% N_2_), MAP2 (20% CO_2_/5% O_2_/75% N_2_), and MAP3 (75% CO_2_/10% O_2_/15% N_2_). The packaged samples were stored at −1 °C. Preservation efficacy was assessed by monitoring changes in microbial counts and physicochemical quality indicators throughout storage. The findings revealed a progressive increase in microbial counts, a deterioration in fish quality, and a darkening of color over extended storage periods. During superchilling storage, the increase in total volatile basic nitrogen (TVB-N) and K value was markedly reduced in the MAP1 group. Regarding protein stability, the MAP1 group exhibited a slower rise in carbonyl content as well as a slower reduction in total sulfhydryl content, further confirming its superior preservation effect. Moreover, this group demonstrated excellence in maintaining the secondary and tertiary structures of myofibrillar proteins, thereby minimizing the structural damage of fish during superchilling storage. In summary, based on observed microbial and protein changes, MAP1 (75% CO_2_/5% O_2_/20% N_2_) was the most effective in preserving quality and extending shelf life.

## 1. Introduction

Half-smooth tongue sole (*Cynoglossus semilaevis*) belongs to a group of large benthic fish species, primarily inhabiting warm temperate marine environments. The muscle tissue of this fish is abundant in excellent protein content and polyunsaturated fatty acids. These substances not only promote muscle growth and development, but also effectively prevent multiple disorders such as hypertension and cardiovascular diseases [1]. However, half-smooth tongue sole is highly susceptible to microbial contamination, which accelerates quality deterioration and shortens its shelf life [2]. Consequently, how to effectively inhibit the growth of microorganisms and prolong the storage shelf life of the half-smooth tongue is a key problem to be solved urgently.

The primary method of creating plasma-activated water (PAW) is to run a plasma generator in water. The principle is that water molecules interact with the particles generated by the plasma generator to produce many active substances. It contains multiple highly reactive components such as O_3_, H_2_O_2_, OH, NO_2_^−^, and NO_3_^−^. They can destroy the cell membrane structure of microorganisms, causing them to lose their barrier function. They can also degrade core macromolecules such as nucleic acids and proteins within the cells, hindering the life activities of microorganisms [3]. PAW has been demonstrated to significantly reduce the activity of spoilage and pathogenic microorganisms in fish, such as *Shigella* and *Pseudomonas*, thereby inhibiting spoilage and extending shelf life [4]. However, the application of PAW still has certain limitations. The main drawback of using PAW in fresh foods is that it tends to promote lipid and protein oxidation, thereby reducing overall product acceptability [5]. Furthermore, the active substances in PAW may gradually degrade, and the sterilization effect may further weaken [6]. In order to enhance its sterilization performance, it is necessary to overcome its limitations and combine PAW with other sterilization techniques.

Modified atmosphere packaging (MAP) is a hurdle technology that extends the shelf life of food by regulating gas composition within the package. It may serve as an ideal complementary technique for PAW. Typically, MAP maintains a low-oxygen environment that significantly inhibits the growth of strict aerobes (especially *Pseudomonas* species) [7]. Studies have shown that the concentration of CO_2_ in the gas mixture must exceed 20–30% in order to significantly reduce microbial growth [8]. Studies have shown that, as the proportion of CO_2_ in MAP increases, the antibacterial effect also gradually strengthens [9]. Li et al. [10] also found that MAP with high CO_2_ content can inhibit the degradation and oxidation of proteins, and reduce the damage to protein structure caused by microorganisms. The MAP technology can provide a specific gas environment, which can not only inhibit the growth of spoilage bacteria but also delay the oxidation of proteins in fish meat, thereby achieving a synergistic preservation effect. Moreover, MAP not only partially compensates for the shortcomings of PAW, but may also exhibit a certain synergistic antibacterial effect when combined with PAW. Therefore, in order to achieve more efficient antibacterial effects, this study mainly employed MAP with high CO_2_ content combined with PAW pretreatment to preserve half-smooth tongue sole.

Additionally, besides packaging and pretreatment, storage temperature significantly impacts the preservation of fish quality. Currently, storage at 4 °C is widely adopted in the aquatic products industry to preserve the original texture and flavor of products. In recent years, superchilling storage has also been validated as an effective method for maintaining fish quality. Superchilling storage refers to a temperature state that lies between conventional cold storage and freezing [11]. Its key advantage is that superchilling storage avoid texture deterioration caused by freezing while maintaining the good quality of the product and a low microbial count [12]. Previous studies have applied superchilling storage to marine products like Atlantic Salmon [13] and Turbot [14]. Furthermore, some reports have indicated that combining appropriate packaging methods with low-temperature preservation techniques effectively extends the shelf life of Atlantic cod filets [15]. Therefore, by integrating superchilling storage with MAP, we could further investigate the effects of multiple preservation methods on the freshness of half-smooth tongue sole.

Although PAW, MAP with high CO_2_, and superchilling have been individually or dually applied in fish preservation, their combined application remains underexplored, especially for half-smooth tongue sole. To enrich the research in this field, this study mainly investigates the effects of PAW pretreatment combined with different packaging methods on the quality of half-smooth tongue sole during superchilling storage, and assesses its preservation efficacy during the storage period. The PAW technology can inactivate surface microorganisms, but its sterilization efficiency is affected by the composition of the food matrix [16]. However, MAP with high CO_2_ inhibits the growth of spoilage bacteria through an anaerobic environment. Compared with traditional freezing preservation technology, the PAW + MAP + superchilling storage combined process has significant cost-effectiveness. It can be integrated into the existing aquatic product processing lines through relevant equipment. While the synergistic effect of both approaches can effectively reduce the risk of microbial contamination in fish meat, further attention is needed regarding their long-term safety implications. Therefore, by integrating the PAW pretreatment, MAP with high CO_2_, and superchilling methods, it is possible to partially overcome the limitations of the above methods and provide a more efficient and sustainable preservation solution for half-smooth tongue sole.

## 2. Materials and Methods

### 2.1. Preparation of PAW and Samples

The preparation of PAW followed the method described by Qian et al. [17]. In this study, PAW was prepared by treating distilled water using a plasma jet device (PG-1000ZD, Sumann Plasma Technology Co., Ltd., Nanjing, China). During PAW preparation, the plasma device operated at 750 W power and 20 kHz discharge frequency. The plasma source was positioned 10 mm above the water surface, and 1 L of distilled water was exposed to the plasma source for 3 min.

The Zhoushan Seafood Market (Zhoushan, China) supplied half-smooth tongue sole, which weighed 1500 ± 50 g each. They were immediately processed and placed on ice for transportation to the laboratory. The uniform fish cubes (150 ± 10 g, 15 × 2.5 × 4 cm^3^) were placed in prepared PAW, soaked for 10 min, and then removed. These treated samples were randomly allocated into 5 groups, which were subjected to distinct packaging protocols: CK (air packaging), VP (vacuum packaging), MAP1 (75% CO_2_/5% O_2_/20% N_2_), MAP2 (20% CO_2_/5% O_2_/75% N_2_), and MAP3 (75% CO_2_/10% O_2_/15% N_2_), respectively. The samples were stored at −1 °C for 60 days, with samples being taken every 5 days. For each sampling, three fish samples per group are selected for water-holding capacity, sensory evaluation, and microbiological analysis. The remaining fish samples are used for physicochemical analysis. All samples underwent triplicate parallel measurements.

### 2.2. Microbiological quality

The detection methods outlined by Li et al. [18] was used to detect the total viable count (TVC), *Pseudomonas* spp. count, psychrophilic bacteria count, and hydrogen sulfide (H_2_S)-producing bacteria count. Both the TVC and psychrophilic bacteria count were determined on plate count agar, with one set being incubated in a 30 °C incubator for 48 h and the other set in a 4 °C refrigerator for 7 d. *Pseudomonas* spp. was cultured on a pseudomonas-selective medium in a 30 °C incubator for 48 h. The H_2_S-producing bacteria were cultured on iron agar in a 30 °C incubator for 48 h. The above-mentioned culture media were all purchased from Qingdao Haibo Biotechnology Co., Ltd. (Qingdao, China). The results were represented as log CFU/g.

### 2.3. Color Difference Determination

The color of the superchilling storage samples was examined with a colorimeter (CR-300, Konica Minolta, Tokyo, Japan). L* (brightness), a* (red-green degree), and b* (yellow-blue degree) were detected to analyze the color changes in the sample.

### 2.4. Water-Holding Capacity (WHC)

The determination of WHC of samples was completed using the procedure described by Xu et al. [19]. An intact fish (3 g) was wrapped in three layers of filter paper and then centrifuged at 8000× *g* for 15 min at 4 °C. WHC represents the mass of the meat after centrifugation divided by the mass before centrifugation. Each group measures three repetitions.

### 2.5. Sensory Evaluation Determination

The sensory evaluation was conducted according to the method described by Shi et al. [20]. The sensory evaluation was conducted using the Quality Index Method (QIM). The evaluation was carried out by 10 sensory experts who had received professional training in accordance with ISO Standard No. 8586: 2023 [21], to ensure the reliability and consistency of the evaluation results. The focus was on examining specific sensory attributes, including odor, color, texture, and overall acceptability. The experts rated these indicators on a 5-point scale, where 5 points indicated excellent, 4 points indicated qualified, 3 points indicated barely qualified, 2 points indicated unqualified, and 1 point indicated poor. The highest score was 5 points, and anything below 2 points was classified as unqualified.

### 2.6. Total Volatile Basic Nitrogen (TVB-N)

The TVB-N value was assayed using the Kjeldahl nitrogen analyzer (Haineng K1100, Hannon, Jinan, China) on a 5 g fish sample, and the obtained results were expressed in the unit of mg N/100 g. Each group measures three repetitions.

### 2.7. K Value Dtermination

The determination of K was carried out using the Waters 2695 high-performance liquid chromatography system, following the method described by Yang et al. [22]. Five grams of fish meat were homogenized with 10 mL of perchloric acid solution (10%, *v*/*v*). The obtained homogenate was centrifuged at 8000× *g* for 10 min at 4 °C, and the supernatant was collected. Then, the precipitate was washed twice with 10 mL of pre-cooled perchloric acid solution (5%, *v*/*v*), and the supernatant was mixed. The pH value of the supernatant was adjusted to the range of 6.0–6.5 using 1 M and 10 M potassium hydroxide solutions. The obtained solution was passed through a membrane with a pore size of 0.22 μm. The filtered samples were stored at −20 °C until further analysis. The K value was calculated according to the following formula:

$$\mathrm{The}\;K\;\mathrm{value}\;(\%)\;=\;\frac{H_{x}R+H_{x}}{ATP+ADP+AMP+IMP+H_{x}R+H_{x}}$$

In the above formula, ATP, ADP, AMP, IMP, H_x_, and H_x_R represent adenosine triphosphate, adenosine diphosphate, adenosine monophosphate, inosine monophosphate, hypoxanthine, and inosine, respectively.

### 2.8. Thiobarbituric Acid Reactive Substances (TBARs)

The extent of lipid oxidation was assessed by determining TBARs levels in accordance with the approach of Yi et al. [23]. Sample (5 g) was homogenized with 25 mL of 20% (*w*/*v*) trichloroacetic acid solution. After standing for 1 h, the homogenate was centrifuged at 8500× *g* for 10 min and filtered. The filtrate was diluted to 50 mL with distilled water, and 5 mL of the diluted filtrate was mixed with 5 mL of 0.02 mol/L thiobarbituric acid (TBA) solution. The mixture was heated in a boiling water bath for 20 min. The reacted solution was cooled, and its absorbance was measured at 532 nm. The calculation formula for the TBA value is presented as follows:

$$\mathrm{TBARs}\;=\;7.8\times A_{532}$$

### 2.9. Low-Field Nuclear Magnetic Resonance (LF-NMR) and Magnetic Resonance Imaging (MRI)

The LF-NMR and MRI assays were performed according to the protocol reported by Wei et al. [24]. The fish (3 cm × 3 cm × 2 cm) was wrapped in polyvinyl chloride wrap. The migration of water in the fish was determined by a LF-NMR equipment (NMI20-040V-I, Niumag, Suzhou, China). The transverse relaxation time (T_2_) was measured using the CPMG sequence, with the parameters set as follows: the number of repeated scans was 2 times, and the number of echoes was 2000 times.

### 2.10. TCA-Soluble Peptide

The determination approach of this indicator refers to that of Pan et al. [25], with a few minor adjustments. Three grams of the fish sample were weighed, and 30 mL of trichloroacetic acid (5%, *w*/*v*) was added to the sample. The mixture was homogenized using a homogenizer and then placed on crushed ice for 30 min. Finally, high-speed centrifugation (10,000× *g*, 5 min) was performed, and the supernatant was collected for the subsequent determination. The soluble peptides in the supernatant were determined by the Lowry protein assay kit.

### 2.11. Myofibrillar Protein (MP) Extraction

The extraction of MPs was based on the method described by Li et al. [26], with slight adjustments. Ten times the volume of Tris–maleate buffer A (0.05 M KCL) was mixed with the fish sample (2 g) to homogenize it. Centrifugation was carried out twice at 10,000× *g* for 15 min to collect the precipitate. Finally, the precipitate was mixed and homogenized with Tris–maleate buffer B (0.6 M KCL), and then centrifuged under the above centrifugal conditions and left to stand for 4 h. After the solution has been left to stand, it was centrifuged to obtain the supernatant, which contained the MPs.

### 2.12. Carbonyl Content and Total Sulfhydryl Content

The levels of thiol and carbonyl groups were both determined by a kit (Beijing Solarbio Technology Co., Ltd., Beijing, China), with the unit being μmol/g. The determination of carbonyl groups is as follows: The MP solution is diluted to 5 mg/mL and then mixed with 2,4-dinitrophenylhydrazine (DNPH). The reaction is carried out at room temperature in the dark for 1 h. Subsequently, trichloroacetic acid is added, and the mixture is centrifuged at 4 °C and 12,000× *g* for 15 min. The supernatant is discarded. The precipitate is washed with a mixed solvent of acetone/ethanol (1:1, *v*/*v*) and centrifuged 3 times at 37 °C, 12,000× *g*, and 4 °C (each 15 min). After the precipitate is completely dissolved, it is centrifuged at 12,000× *g* for 10 min, and the supernatant is discarded. The absorbance at 370 nm is measured. The determination of sulfhydryl content is as follows: The concentration of MP solution is diluted to 5 mg/mL, and then mixed with Tris-Gly buffer and thoroughly mixed. Subsequently, a quantitative DTNB solution is added and thoroughly mixed, and the reaction is carried out at 4 °C in the dark. After the reaction, the mixture is centrifuged (3000× *g*, 10 min). The absorbance of supernatant at 412 nm is measured.

### 2.13. MPs Secondary Structure Determination

The secondary structure of MPs was measured by a Jasco J-1500 circular dichroism chromatograph (Jasco, Tokyo, Japan). The scanning wavelength range was set at 180 to 260 nm, with an interval of 1 nm. The optical path was fixed at 1 mm, and the final value was taken as the average of three consecutive measurements.

### 2.14. MPs Tertiary Structure Determination

The tertiary structure of MPs was analyzed with a fluorescence spectrophotometer (F-7100, Hitachi, Tokyo, Japan) in accordance with the method of Yu et al. [27].

### 2.15. Statistical Analysis

The experimental data were analyzed using the IBM SPSS 27.0 software (version 27.0, SPSS Inc., Chicago, IL, USA). A one-way analysis of variance (ANOVA)–Duncan test was employed to determine significant differences (*p* < 0.05). Each experiment was conducted with three replicates, and the results were presented as mean ± standard deviation.

## 3. Results and Discussion

### 3.1. Microbial Analysis

#### TVC

**Figure 1:**
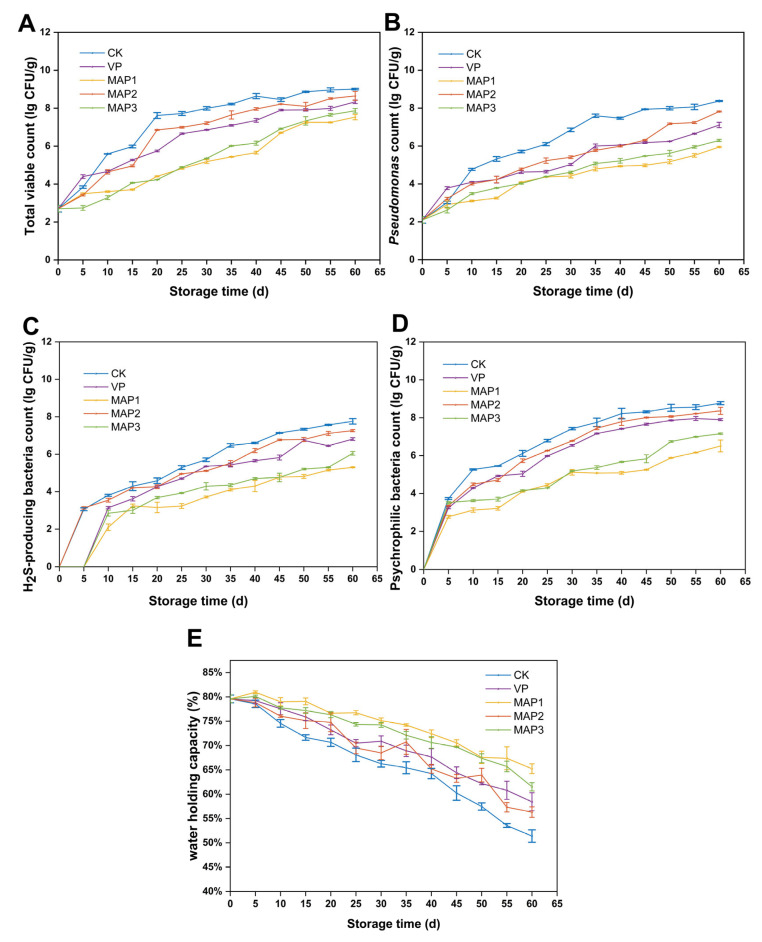
Changes in microbial levels of half-smooth tongue sole under different packaging conditions during superchilling storage. (**A**) TVC; (**B**) *Pseudomonas* spp. count; (**C**) H_2_S-producing bacteria count; (**D**) psychrophilic bacteria count; (**E**) WHC. Bars represent standard deviation (*n* = 3).

Fish are susceptible to contamination by various microorganisms during storage. Therefore, observing the trend of TVC changes during superchilling storage is crucial. The initial TVC of fish was 2.70 log CFU/g. In the course of superchilling storage, the TVC gradually increased in all groups (*p* < 0.05) (Figure 1A). The CK group exhibited a faster growth rate, exceeding the limit (7.0 log CFU/g) on the 20th day, demonstrating that the fish were spoiled. This value exceeds the maximum acceptable limit stipulated by international food microbiological standards (5.0 log CFU/g); therefore, these fish were no longer suitable for human use and consumption [28]. However, the microbial growth in the MAP1 and MAP3 groups was relatively slow, while that in the MAP2 group was relatively fast. The possible reason is the antibacterial effect of the high content of CO_2_ in MAP. Studies also show that a CO_2_ level above 20% can inhibit spoilage bacteria such as *Pseudomonas*, and when the level reaches 50%, acidifying the surface of the fish can enhance the inhibitory effect, creating unfavorable conditions for microbial growth [18]. When the CO_2_ content is high, it causes a decrease in the pH of the system. The decrease in pH leads to the partial depolarization of the microbial cell membrane, allowing CO_2_ to penetrate the microbial cell membrane and disrupting the pH homeostasis within the cell [29]. Moreover, changes in intracellular pH can affect the physiological processes of the cell (such as cell proliferation, differentiation, and apoptosis, as well as cell signal transduction) [30]. Additionally, the disruption of cell membrane integrity may result in the loss of membrane potential, thereby weakening proton power and affecting ATP synthesis and cellular metabolism [31]. Therefore, MAP1, due to its high proportion of CO_2_, can effectively inhibit the growth of bacteria.

Psychrophilic bacteria, due to their adaptability to low-temperature environments, also pose a certain threat to fish during superchilling storage. No psychrophilic bacteria were detected during the initial superchilling storage phase (Figure 1D), likely due to PAW inhibiting their growth. Sun et al. [32] also found that 20, 40, and 60 min PAW treatments reduced psychrophilic bacteria by 0.11, 0.75, and 0.56 log CFU/g, respectively. The growth trends of psychrophilic bacteria across all groups during superchilling storage largely mirrored those of TVC. Furthermore, the figure indicates that MAP1 and MAP3 exhibited slower microbial growth increases. This suggests that high CO_2_ concentrations inhibited psychrophilic bacterial growth, a conclusion that aligns with the research results of Babic Milijasevic et al. [33].

Most spoilage during superchilling storage is caused by microorganisms, such as *Pseudomonas* spp. and H_2_S-producing bacteria. Initial superchilling storage showed negligible *Pseudomonas* and H_2_S-producing bacteria counts, indicating their minimal proportion in total microbial populations. Throughout the entire storage process, both *Pseudomonas* and H_2_S-producing bacteria counts steadily increased (Figure 1B,C). The CK group exhibited faster growth, reaching limit values for both counts on the 35th and 45th day. The high-CO_2_ groups (MAP1 and MAP3) showed markedly slower microbial growth increases, failing to reach the limit even by the end of storage. Studies indicate that, in oxygen-depleted environments with CO_2_, aerobic bacteria are significantly inhibited, thereby suppressing Gram-negative bacteria including *Pseudomonas* spp. and H_2_S-producing bacteria. Throughout the storage process, *Pseudomonas* counts remained relatively higher than H_2_S-producing bacteria counts, likely due to *Pseudomonas* inhibiting the growth of hydrogen sulfide producers. As a significant food spoilage bacterium, research indicates that *Pseudomonas* produces large amounts of iron carriers during spoilage, chelating iron in the environment to enhance its own competitiveness and thereby suppressing H_2_S-producing bacteria [34].

### 3.2. Physical and Chemical Analyses

#### 3.2.1. Color Difference

Color is widely regarded as a crucial quality attribute of meat, directly influencing consumer choice. During superchilling storage, the L* values of all samples exhibited a progressive decline. The MAP groups (MAP1 and MAP3) exhibited higher L* values than the CK, VP, and MAP2 groups (Table 1). The variation in L* values may stem from changes in fish moisture content during storage. Ramadhan et al. [35] similarly attributed L* shifts to moisture loss during storage, which reduces surface brightness and consequently alters L* values. As the storage time increases, the color change in meat is related to the oxidation of myoglobin to metmyoglobin, which may be due to the decrease in the reductivity of metmyoglobin, resulting in the accumulation of metmyoglobin in the meat [36]. Oxymyoglobin as one type of myoglobin may also be affected by bacterial growth or oxygen tension levels during the transition to metmyoglobin [37,38]. Oxygen in food acts as an oxidizing agent, promoting the formation of metmyoglobin [39]. Therefore, in the CK group, due to the accumulation of metmyoglobin, the color is darker and L* is lower. Zduńczyk et al. [40] also stated that the presence of CO_2_ in packaging may lead to acidification, causing protein denaturation, resulting in changes in the structure of MPs and increased light scattering. Therefore, the L* value in the MAP is higher. As presented in Table 1, the a* values of fish in all groups gradually decreased while b* values gradually increased, indicating the fish progressively turned yellow. It might be due to the presence of oxygen that myoglobin and lipids in fish meat are oxidized, resulting in a weakening of red color and a gradual shift towards yellow [40].

#### 3.2.2. WHC

WHC represents the ability of muscle proteins to prevent water release when subjected to external forces. Throughout the storage, the WHC of fish in all treatment groups significantly decreased (*p* < 0.05) (Figure 1E). At the completion of superchilling storage, the WHC values for the CK, VP, MAP1, MAP2, and MAP3 groups were 51.4%, 58.4%, 65.2%, 56.3%, and 61.5%, respectively. The decrease in WHC during superchilling storage may result from protein oxidation. Studies have shown that protein oxidation can induce cross-linking reactions among molecules, resulting in changes in the distance between myofilaments [41]. During this process, it causes water loss in the muscle, ultimately reducing its water retention capacity. The CK group experienced significant water loss during storage because it failed to inhibit microbial growth and protein oxidation. During superchilling storage, the WHC of MAP1 and MAP3 decreased more slowly, particularly in the MAP1 group. This demonstrates that MAP with low oxygen and high CO_2_ ratios can delay the decline in WHC [14]. The results of this study demonstrated that microbial growth triggered the accumulation of insoluble protein aggregates and protein denaturation, which in turn impaired the structural integrity of proteins [42]. The low oxygen and CO_2_ gas composition within MAP inhibits bacterial growth and metabolism, slowing the degradation and denaturation of fish proteins [43]. This facilitates a tighter binding between water and proteins, thereby effectively stabilizing the muscle’s capacity to retain moisture.

#### 3.2.3. Sensory Evaluation

**Figure 2:**
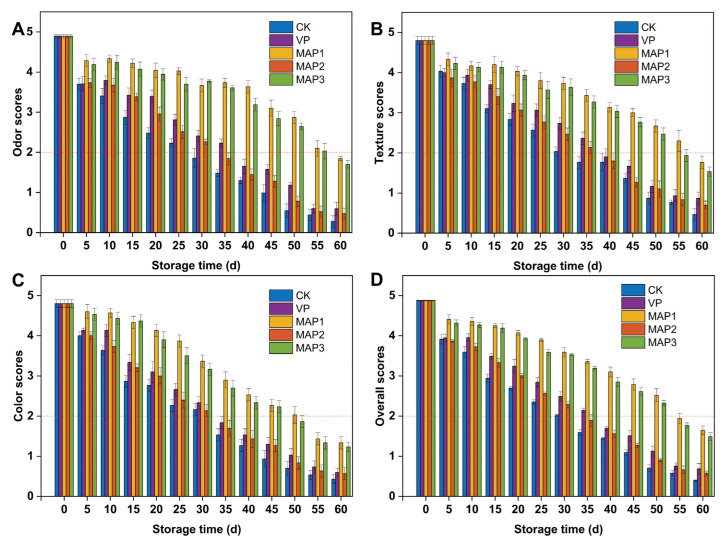
Changes in sensory evaluation ((**A**) odor scores, (**B**) texture scores, (**C**) color scores, and (**D**) overall scores) under different packaging conditions during superchilling storage. Bars represent standard deviation (*n* = 3).

Appearance, texture, taste, and overall acceptability are key factors determining the final quality of food. Figure 2 illustrates the effects of different treatments on the sensory attributes of half-smooth tongue sole (including odor, texture, color, and overall acceptability). Scores above 2 are considered acceptable. As storage time increased, sensory scores gradually declined across all groups. The CK group’s sensory score reached 2 on 30th day, becoming unacceptable. The VP and MAP2 groups deviated from 2 on 40th day, becoming unacceptable. Moreover, compared to microbial and chemical results, the quality assessments of superchilled fish samples exhibited a certain delay. This may stem from the subjective nature of sensory evaluation. The deterioration of the fish quality may occur internally first, and it can be visually detected later [44]. The MAP1 and MAP3 groups only fell below 2 toward the end of storage. This may be related to the high CO_2_ levels inhibiting microbial growth. Research indicates that, during storage, spoilage bacteria produce characteristic odor- and flavor-altering metabolites, such as amines, sulfides, and aldehydes [45]. They significantly degrade sensory quality. Texture deterioration primarily results from the combined effects of microbial growth and endogenous enzymes, which disrupt the tertiary structure of MPs [46]. Color deterioration is mainly attributed to the water migration and oxidative degradation of muscle components, which subsequently lowers odor scores [47]. MAP with high CO_2_ and low oxygen suppresses protein oxidation and the formation of metmyoglobin, thereby stabilizing the color of the meat [48]. MAP with high CO_2_ ratio may also inhibit bacterial growth and metabolism, reducing sulfur compounds and ammonia produced during metabolic processes and thereby minimizing off-flavor formation [33].

#### 3.2.4. TVB-N Value

**Figure 3:**
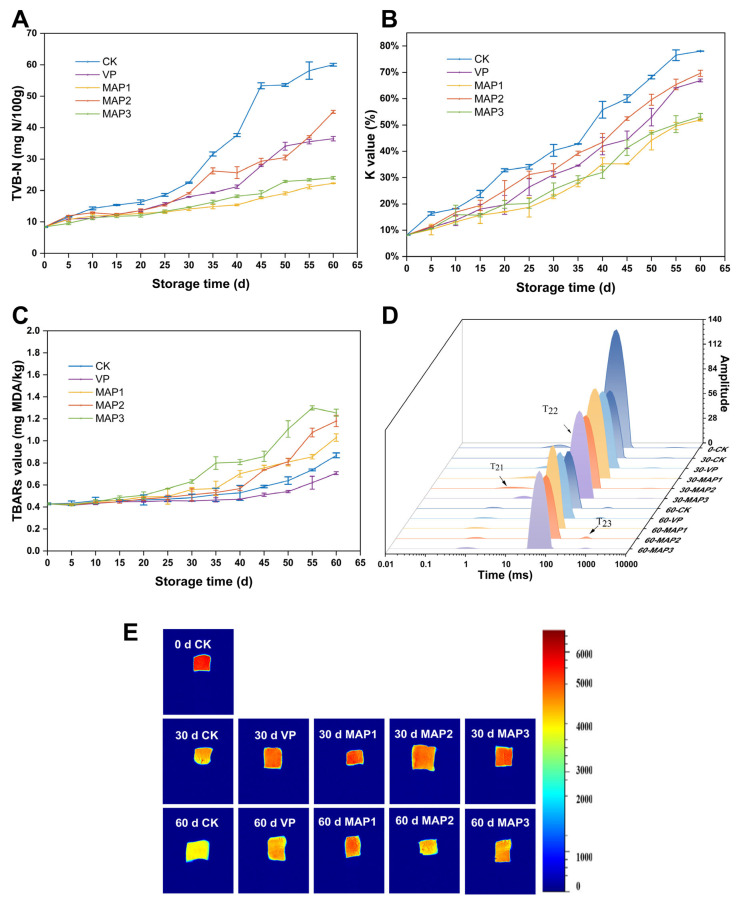
Changes in (**A**) TVB-N, (**B**) K value, (**C**) TBARs, (**D**) LF-NMR, and (**E**) MRI under different packaging conditions during superchilling storage. Bars represent standard deviation (*n* = 3).

TVB-N is a nitrogen compound formed by the combination of basic nitrogen substances produced during the decomposition of fish protein with organic acids. Changes in its content reflect the spoilage process of aquatic products [49]. At the beginning of superchilling storage, the TVB-N value of fish was 8.43 mg N/100 g (Figure 3A). Protein degradation is primarily influenced by endogenous enzymes and microbial activity. Studies also indicate that bacteria can proliferate by metabolizing amino acids produced during protein degradation, leading to rapid increases in TVB-N and other decarboxylation products [50]. During the entire superchilling period, TVB-N values rose across all samples, mirroring the trend in the TVC (*p* < 0.05). The figure indicates a relatively slow increase in TVB-N during the early stage, accelerating progressively in the middle stage. This finding primarily stems from superchilling storage inhibiting bacterial growth and reproduction, thereby reducing volatile nitrogen compounds produced by protein degradation due to microbial activity during the early stage [51]. The MAP2 group displayed a more rapid increase in TVB-N, most likely because of the low CO_2_ levels favored aerobic bacterial growth. This promoted microbial proliferation, protein hydrolysis, and endogenous enzyme activity, further accelerating spoilage and increasing TVB-N levels [49]. In contrast, fish stored under MAP with low oxygen and high CO_2_ ratios maintained TVB-N levels under the limit on the final day of storage. This is similar to the findings reported by Babic et al. [33]. MAP may also influence autolysis and muscle proteolysis rates during storage by delaying lysosomal degradation and reducing the release of lysosomal enzymes [42]. Furthermore, CO_2_ dissolved into the fish within MAP forms carbonic acid, which further inhibits other microorganisms, thereby preventing the microbial degradation of proteins [52].

#### 3.2.5. K Value

K value is frequently utilized to assess fish freshness. An increase in K value indicates the degree of nucleotide degradation in fish. This also reflects the build-up of ATP breakdown products: uridine monophosphate and hypoxanthine [53]. Nucleotide degradation is primarily influenced by endogenous autolytic enzyme activity and microbial action [54]. With extended storage duration, all samples showed a considerable rise in K values (*p* < 0.05) (Figure 3B), which is in line with the outcomes reported by Tian et al. [55]. At the beginning of superchilling storage, the K value of the fish was 8.16%. On the 45th day, the K value in the CK group exceeded the specified limit (60%), indicating spoilage had occurred. The VP and MAP2 groups also exhibited spoilage on the 55th day and 50th day, respectively. Research indicates that the degradation of inosine 5′-monophosphate and its related products is closely associated with microorganisms, primarily driven by various enzymes (such as 5′ nucleotide enzymes) produced by these microorganisms [56]. Furthermore, the degradation of ATP during storage is the result of the combined action of multiple enzymes. Since the CK, VP, and MAP2 groups failed to effectively inhibit microbial growth, the enzymes produced by microorganisms may have promoted ATP degradation, leading to increased K values. However, by the end of superchilling storage, the MAP1 and MAP3 groups remained below the threshold, with the MAP1 group exhibiting particularly slow increases throughout storage. Chen et al. [57] similarly reported that a high concentration of CO_2_ inhibited microbial growth and the associated enzyme secretion, suggesting that MAP with low oxygen and a high level of CO_2_ effectively delays the K value increase rates.

#### 3.2.6. TBARs

The TBARs value, determined by the malondialdehyde content, serve as a core indicator for assessing the degree of lipid oxidation in seafood. These compounds cause unpleasant and rancid odors, making TBARs a useful measure for assessing product freshness [58]. As shown in Figure 3C, no noticeable variations in TBARs were observed among all groups around the early storage phase (*p* > 0.05). The TBARs value significantly increased between groups in the later phase (*p* < 0.05). This might be potentially because the early storage phase represents the induction period for fatty acid degradation [52]. The TBA values increased more rapidly in the MAP group, while the TBARs in the VP group showed minimal change, which is in agreement with the conclusions of Yin et al. [59]. This could be ascribed to the absence of oxygen in the vacuum group, which prevented aerobic bacteria from growing and metabolizing and effectively slowed the oxidation and rancidity of lipids during storage [60]. However, at the last stage of superchilling storage, the MAP2 group achieved higher TBARs than the MAP1 group. This phenomenon may result from high CO_2_ concentrations inhibiting microbial growth and lipase release, thereby slowing TBARs accumulation [61]. Among all the groups, the TBARs value of the control group CK was the lowest, while the TBARs values were the highest in the different MAP groups (MAP1, MAP2, and MAP3). This is consistent with the findings of Pastoriza et al. [62]. The possible reason is that the CO_2_-modified gas proportion is prone to form carbonic acid, which leads to a change in the conformation of the protein. This process releases free heme iron [63]. Free heme iron has also been proven to be a potential pro-oxidant in the muscle system, and its release may accelerate lipid oxidation in the muscle [64]. In addition, MAP may stabilize the active substances from the pretreatment with plasma in the early stage. The active substances further promote lipid oxidation. Olatunde et al. [65] found that, regardless of the gas composition inside the packaging bag, the TBARs of the samples after plasma treatment were higher. The relatively low TBARS values observed in the CK group may be attributed to the direct utilization of malondialdehyde and other TBARS components by microorganisms, or the reaction of these TBARS with amine compounds generated during bacterial metabolism [66]. Therefore, the packaging rich in CO_2_ effectively inhibits microbe-induced spoilage, but it cannot prevent chemical deterioration, especially lipid oxidation. Considering combined TVB-N, K value, and TBARs results, the MAP1 group effectively extends the shelf life of fish.

#### 3.2.7. LF-NMR and MRI

LF-NMR effectively evaluates water mobility and distribution within all fish samples. T_21_ depicts water firmly attached to macromolecules (e.g., proteins) via molecular forces, while T_22_ represents water confined inside the extremely ordered MP matrix [67]. T_23_ signifies free water, which is readily removable. While T_22_ showed a declining tendency throughout the superchilling storage process, T_21_ showed minimal variation with little overall change (Figure 3D). T_23_ demonstrated an increasing trend from the initial stage to the end of storage, and this trend is the same as that in the experimental findings of Pan et al. [68]. This trend could be explained by the destruction of MP structure during storage, which converts bound water into free water, contributing to a reduction in the peak value of T_22_ and an increase in the peak value of T_23_ [69]. The T_22_ peak areas in the MAP1 and MAP3 groups were substantially higher than those in the CK, VP, and MAP2 groups, demonstrating that water within the MAP fish was more tightly and stably bound to macromolecules. Studies also suggest the MAP inhibitory effect on fish moisture stems from suppressing bacterial degradation and the disruption of myofibrillar proteins.

MRI serves as a method for visualizing the internal structure of fish, where red indicates high proton density and blue denotes low proton density. In MRI images, when the hydrogen proton density increases, the pixel signal intensity in the proton density-weighted image of the sample also becomes correspondingly stronger, which also indicates that the moisture content of the sample is relatively high [70]. During storage, proton images showed that the red areas of all samples gradually decreased and shifted towards yellow (Figure 3E), indicating moisture loss and deteriorating quality in fish [67]. Furthermore, proton density changes more rapidly in the CK, VP, and MAP2 groups. This indicates that the MAP1 and MAP3 groups better maintain the fish moisture content.

#### 3.2.8. TCA-Soluble Peptides

**Figure 4:**
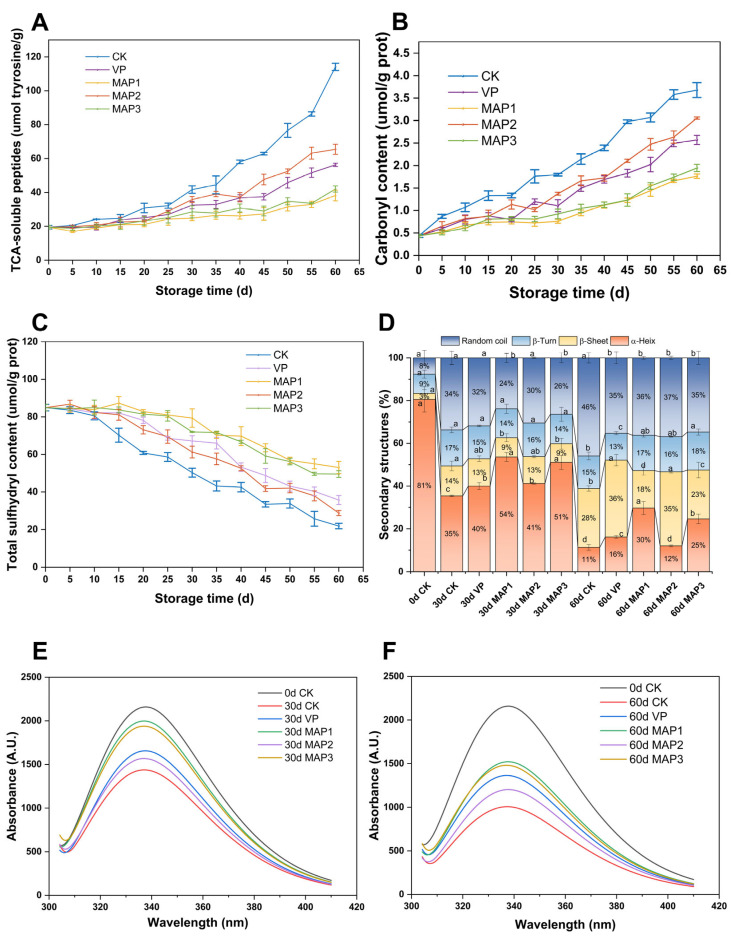
Changes in (**A**) TCA-soluble peptides, (**B**) carbonyl content, (**C**) total sulfhydryl content, (**D**) secondary structure of MPs, and (**E**,**F**) tertiary structure of MPs under different packaging conditions during superchilling storage. Different letters (a–d) show a statistically significant difference (*p* < 0.05). Bars represent standard deviation (*n* = 3).

TCA-soluble peptides represent the extent of protein hydrolysis and are utilized as a measure of small-molecule peptide content. Fresh samples exhibited a TCA-soluble peptide level of 19.46 μmol tyrosine/g. All five sample groups showed an increasing trend in TCA-soluble peptides, with no discernible variations among the groups during the first 25 days of storage (*p* > 0.05) (Figure 4A). However, after 25 days, the CK group had significantly higher levels than the other experimental groups (*p* < 0.05). The slower increase in TCA-soluble peptides during early storage may result from the storage temperature (−1 °C) in this experiment inhibiting the endogenous protease activity in fish, thereby delaying TCA-soluble peptide accumulation [71]. Research indicates that bacteria create proteases largely during their late exponential development phase. Therefore, the marked increase in TCA-soluble peptides during the later phases of storage may be attributed to the elevated levels of proteases and peptidases secreted by microorganisms [72]. The increase in TCA-soluble peptides in the MAP1 and MAP3 groups was markedly slower (*p* < 0.05), indicating that a high proportion of CO_2_ can delay the hydrolysis of MPs.

#### 3.2.9. Carbonyl Content

Protein oxidation produces carbonyl compounds, which can be measured to determine the extent of protein oxidation [73]. As shown in Figure 4B, myofibrillar protein carbonyl content steadily increased throughout storage, with the CK group experiencing the fastest increase. Samples under MAP exhibited lower carbonyl accumulation and slower oxidation rates (*p* < 0.05), indicating that MAP can inhibit MP oxidation. Other studies have also shown that meat stored in MAP exhibits slower oxidation rates, primarily due to reduced oxygen levels, increased carbon dioxide supply, the inhibition of microbial growth, and the decreased activity of oxidase enzymes (such as lipoxygenase and epoxidase) that promote meat oxidation [74]. Increased carbonyl content leads to protein cross-linking, affecting protein structure and reducing muscle water retention capacity [75]. Furthermore, protein oxidation promotes increased disulfide bond formation, leading to MP aggregation and the disruption of protein structural integrity [76]. MAP with high CO_2_ ratio inhibits protein oxidation, reducing both carbonyl accumulation and thiol exposure while minimizing disulfide bond formation, thereby maintaining protein structural stability. Furthermore, MAP2 exhibited faster carbonyl accumulation compared to MAP1 and MAP3, indicating that CO_2_ levels above 20% are required for effective oxidation inhibition [77].

#### 3.2.10. Total Sulfhydryl Content

Thiols represent the most reactive functional groups in fish proteins, and variations in their content signal the level of oxidative denaturation in MPs [78]. The initial total sulfhydryl content of fresh samples was 83.58 μmol/g prot. During superchilling storage, the sulfhydryl content gradually decreased in all samples, with the CK group showing the greatest loss (*p* < 0.05), while the other groups exhibited relatively slower changes (Figure 4C). This indicates that oxidation occurred in all fish samples throughout storage. The quick reduction in sulfhydryl groups in the CK group likely resulted from MPs being exposed to air, causing protein oxidation. Additionally, studies have shown that free radicals are produced during lipid oxidation. These free radicals not only attack sulfhydryl groups but also cause oxidative damage to MPs [79,80]. The total sulfhydryl content in the MAP1 group showed the slowest change, possibly because the MAP with high CO_2_ levels not only restricted reactive oxygen species formation but also inhibited microbial activities and enzyme activities, thereby slowing down MP oxidation [81]. During storage, exposed sulfhydryl groups are prone to oxidation, forming disulfide bonds within or between peptides [82]. High CO_2_ levels in MAP inhibit protein oxidation, reducing the formation of disulfide bonds and thereby slowing the decline in sulfhydryl content.

#### 3.2.11. The Secondary Structure of MPs

Figure 4D shows the secondary structure of MPs: α-helix, β-fold, β-turn, and random curl. The α-helix maintains its stable shape through intramolecular hydrogen bonding between carbonyl oxygen and amino hydrogen in the polypeptide chain [83]. Typically, higher β-angle and disordered coiling fractions indicate greater instability and looseness in protein structure [42,84]. A significant reduction in α-helix and a rise in β-folding content are shown in Figure 4D, indicating that the structure of MP has undergone a transformation. According to studies, the breakdown of hydrogen bonds during the catalytic process of protein degradation by microbial enzymes can speed up the change in proteins’ secondary structures into disordered structures [18]. The CK, VP, and MAP2 groups exhibited less α-helices and more random curls, while the MAP1 and MAP3 groups indicated more α-helices and less random curls. Research has found that high CO_2_ concentrations can penetrate microbial cell membranes, leading to cytoplasmic acidification that impairs enzyme activity and disrupts cellular metabolism [85]. Furthermore, CO_2_ serves as a metabolic byproduct or cofactor in numerous biochemical reactions, significantly altering enzyme activity and cellular metabolism [86]. Consequently, elevated CO_2_ may also inhibit microbial enzyme activity, thereby reducing the degradation of MP structures by enzymes produced by microorganisms. Protein oxidation induces carbonylation, protein aggregation, and disulfide bond disruption, leading to protein cross-linking and compromising protein integrity [42,75]. High concentrations of CO_2_ can create a low-oxygen environment, thereby reducing protein oxidation and stabilizing the structure of MPs.

#### 3.2.12. The Tertiary Structure of MPs

Endogenous fluorescence spectroscopy can be used to study the tertiary structural changes in proteins by identifying tryptophan residues in the MP structure [87]. The tryptophan fluorescence intensity of all samples gradually weakened over time and reached the lowest value on the 60th day (Figure 4E,F). Studies have shown that oxidative induction can lead to the swelling of MP structures, thereby exposing tryptophan residues and reducing the fluorescence intensity of tryptophan [88]. During the entire preservation period, the fluorescence intensities of the CK, VP, and MAP2 groups decreased significantly. However, the MAP1 and MAP3 groups consistently had higher fluorescence intensities than the other groups. High CO_2_ levels inhibit microbial activity, thereby reducing the degradation of MPs by exogenous microbial enzymes during extended storage and preserving their integrity [18]. Research indicates that protein oxidation promotes conformational unfolding, exposing aromatic amino acid residues originally located within the molecular interior of the protein surface [42]. This subsequently triggers protein cross-linking, aggregation, and denaturation. Consequently, elevated CO_2_ levels may also inhibit protein oxidation, thereby maintaining the stability of tertiary structures. Therefore, the MAP1 and MAP3 groups stabilized the tertiary structure of MPs. Combining the indicators of MP changes, the MAP1 group can effectively slow down the degradation of proteins.

## 4. Conclusions

This study aims to evaluate the preservation effect of PAW pretreatment combined with different MAP conditions on half-smooth tongue sole during superchilling storage, and to evaluate the impact of this composite technology on fish quality and protein stability. The results of this study indicate that using PAW pretreatment combined with MAP1 packaging (75% CO_2_/5% O_2_/20% N_2_) can delay the deterioration of fish quality and protein stability during superchilling storage. During the final period of superchilling storage, the total quantity of colonies, the content of TVB-N and TCA-soluble peptides, and the K value of the MAP1 group remained at relatively low levels. In terms of protein stability, during the superchilling storage, the carbonyl and sulfhydryl group content in the MAP1 group changed from 0.44 μmol/g prot and 84.93 μmol/g prot on 0 day to 1.76 μmol/g prot and 53.03 μmol/g prot, respectively. Compared to other treatment groups, these changes occurred relatively slowly, further confirming the superior preservation effect of MAP1. This group performed outstandingly in maintaining the secondary and tertiary structures of MPs, leading to relatively minor structural alterations in the fish during superchilling storage. In summary, these findings confirm that the synergistic effects of PAW pretreatment, MAP1, and superchilling storage play a crucial role in maintaining the long-term storage quality of half-smooth tongue sole. This combination could be applied industrially to extend the shelf life of fish products. One potential drawback and limitation of this study is that the MAP with high CO_2_ might cause the packaging to collapse, affecting the appearance of the products. Additionally, some samples may develop a slight sour taste during long-term storage.

## Figures and Tables

**Table 1:** Changes in color difference in half-smooth tongue sole under different packaging conditions during superchilling storage.

**Index**		L*				
**Treatment**		**CK**	**VP**	**MAP1**	**MAP2**	**MAP3**
Change in color	0 d	64.35 ± 0.33 a	64.35 ± 0.33 a	64.35 ± 0.33 a	64.35 ± 0.33 a	64.35 ± 0.33 a
	5th day	65.03 ± 3.04 bc	66.9 ± 0.22 ab	66.47 ± 0.04 abc	64.14 ± 0.15 c	68.54 ± 0.06 a
	10th day	60.16 ± 0.04 b	63.43 ± 1.26 a	59.72 ± 0.17 b	60.98 ± 0.4 b	63.54 ± 1.36 a
	15th day	58.12 ± 0.06 c	57.29 ± 0.29 d	59.93 ± 0.18 b	59.99 ± 0.23 b	62.76 ± 0.32 a
	20th day	56.74 ± 0.26 d	56.44 ± 0.25 d	58.11 ± 0.15 b	57.2 ± 0.14 c	61.72 ± 0.03 a
	25th day	55.85 ± 0.74 cd	55.39 ± 0.4 d	57.72 ± 0.07 b	56.58 ± 0.51 c	60.31 ± 0.02 a
	30th day	54.36 ± 0.48 d	55.49 ± 0.15 c	59.96 ± 0.23 a	55.39 ± 0.12 c	57.2 ± 0.38 b
	35th day	53.32 ± 0.37 d	54.42 ± 0.32 c	56.11 ± 0.54 ab	55.65 ± 0.47 b	56.39 ± 0.13 a
	40th day	44.67 ± 0.2 c	53.2 ± 0.85 b	55.74 ± 0.06 a	53.13 ± 0.39 b	55.46 ± 0.16 a
	45th day	52.46 ± 5.32 b	46.37 ± 0.93 c	57.39 ± 2.24 a	45.6 ± 0.07 c	55.96 ± 0.22 ab
	50th day	46.17 ± 0.14 d	49.81 ± 0.46 b	59.05 ± 0.35 a	39.84 ± 0.83 e	48.05 ± 0.71 c
	55th day	38.96 ± 0.6 c	31.02 ± 0.77 d	49.82 ± 0.07 a	21.84 ± 1.93 e	44.13 ± 1.18 b
	60th day	38.87 ± 1.01 c	46.42 ± 1.91 a	45.7 ± 0.48 ab	39.26 ± 1.19 c	44.25 ± 0.1 b
**Index**	a*					
**Treatment**		**CK**	**VP**	**MAP1**	**MAP2**	**MAP3**
Change in color	0 d	−3.75 ± 0.07 a	−3.75 ± 0.07 a	−3.75 ± 0.07 a	−3.75 ± 0.07 a	−3.75 ± 0.07 a
	5th day	−2.8 ± 0.06 a	−2.86 ± 0.16 a	−2.82 ± 0.21 b	−2.83 ± 0.2 ab	−2.85 ± 0.23 c
	10th day	−2.46 ± 0.21 a	−2.67 ± 0.17 b	−2.72 ± 0.17 ab	−2.79 ± 0.06 b	−2.64 ± 0.2 ab
	15th day	−1.92 ± 0.76 a	−1.85 ± 0.73 a	−1.81 ± 0.67 b	−2.2 ± 0.19 ab	−2.76 ± 0.79 ab
	20th day	−2.14 ± 0.03 a	−2.45 ± 0.52 c	−2.69 ± 0.47 c	−2.98 ± 0.1 b	−3.04 ± 0.18 d
	25th day	−3 ± 0.17 a	−2.98 ± 0.15 a	−3.04 ± 0.04 a	−3.05 ± 0.05 a	−3.09 ± 0.07 b
	30th day	−4.09 ± 0.15 c	−3.95 ± 0.39 ab	−3.65 ± 0.43 a	−3.34 ± 0.15 b	−3.13 ± 0.23 a
	35th day	−4.2 ± 0.06 c	−4.1 ± 0.21 b	−4.02 ± 0.22 a	−3.77 ± 0.21 b	−3.52 ± 0.39 a
	40th day	−4.65 ± 0.04 c	−4.54 ± 0.2 b	−4.36 ± 0.31 a	−4.16 ± 0.14 a	−3.89 ± 0.33 a
	45th day	−4.99 ± 0.14 b	−4.68 ± 0.41 a	−4.56 ± 0.35 a	−4.44 ± 0.2 b	−4.35 ± 0.35 a
	50th day	−4.98 ± 0.2 c	−4.86 ± 0.03 bc	−4.89 ± 0.06 a	−4.91 ± 0.06 b	−4.7 ± 0.41 a
	55th day	−6.77 ± 0.39 b	−6.45 ± 0.51 b	−6.37 ± 0.37 a	−6.3 ± 0.4 b	−5.83 ± 1.11 a
	60th day	−7.3 ± 0.34 c	−7.02 ± 0.2 b	−6.95 ± 0.23 a	−6.81 ± 0.01 bc	−6.58 ± 0.39 a
**Index**	b*					
**Treatment**		**CK**	**VP**	**MAP1**	**MAP2**	**MAP3**
Change in color	0 d	−6.39 ± 0.09 a	−6.39 ± 0.09 a	−6.39 ± 0.09 a	−6.39 ± 0.09 a	−6.39 ± 0.09 a
	5th day	−6.39 ± 0.09 e	−4.48 ± 0.19 c	−3.5 ± 0.32 a	−5.49 ± 0.12 d	−4.1 ± 0.29 b
	10th day	−4.58 ± 0.38 c	−2.84 ± 0.23 a	−3.69 ± 0.22 b	−4.58 ± 0.38 c	−3.9 ± 0.46 b
	15th day	−3.85 ± 0.09 d	−2.27 ± 0.05 a	−3.68 ± 0.06 c	−2.95 ± 0.13 b	−3.07 ± 0.07 b
	20th day	−3.61 ± 0.09 c	−1.71 ± 0.33 a	−3.8 ± 0.09 c	−2.97 ± 0.51 b	−2.64 ± 0.06 b
	25th day	−3.39 ± 0.47 c	−1.44 ± 0.06 ab	−1.24 ± 0.06 a	−1.73 ± 0.11 b	−1.29 ± 0.14 a
	30th day	−1.49 ± 0.44 b	−1.08 ± 0.09 b	−0.39 ± 0.19 a	−1.59 ± 0.57 b	−0.95 ± 0.21 ab
	35th day	0.42 ± 0.07 b	−0.95 ± 0.19 c	−0.48 ± +0.16 c	−0.71 ± 0.08 c	2.62 ± 0.77 a
	40th day	0.53 ± 0.08 c	2.01 ± 0.05 a	1.44 ± 0.3 b	1.57 ± 0.32 b	1.98 ± 0.19 a
	45th day	2.94 ± 0.52 ab	2.28 ± +0.16 b	2.52 ± 0.22 ab	3.08 ± 0.16 a	3.09 ± 0.54 a
	50th day	3.34 ± 0.18 b	4.57 ± 0.66 a	4.41 ± 0.54 a	4.19 ± 0.08 a	4.67 ± 0.24 a
	55th day	4.21 ± 0.53 c	5.44 ± 0.13 b	6.53 ± 0.32 a	4.46 ± 0.93 c	6.07 ± 0.34 ab
	60th day	5.43 ± +0.26 d	7.67 ± 0.24 b	8.52 ± 0.27 a	6.11 ± 0.18 c	7.87 ± 0.5 b

All data are uniformly expressed as mean ± standard deviation with letters a–e denoting significant differences between groups (*p* < 0.05, *n* = 3).

## Data Availability

The original contributions presented in the study are included in the article, and further inquiries can be directed to the corresponding authors.
